# Systemic inflammation, blood-brain barrier vulnerability and cognitive/non-cognitive symptoms in Alzheimer disease: relevance to pathogenesis and therapy

**DOI:** 10.3389/fnagi.2014.00171

**Published:** 2014-07-29

**Authors:** Shuko Takeda, Naoyuki Sato, Ryuichi Morishita

**Affiliations:** ^1^Department of Clinical Gene Therapy, Graduate School of Medicine, Osaka University, YamadaokaSuita, Osaka, Japan; ^2^Department of Geriatric Medicine, Graduate School of Medicine, Osaka University, Yamada-okaSuita, Osaka, Japan

**Keywords:** systemic inflammation, blood-brain barrier, Alzheimer disease, pathogenesis, therapy

## Abstract

The incidence of dementia is increasing at an alarming rate, and has become a major public health concern. Alzheimer disease (AD) is the most common form of dementia and is characterized by progressive cognitive impairment. In addition to classical neuropathological features such as amyloid plaques and neurofibrillary tangles (NFT), accumulation of activated immune cells has been documented in the AD brain, suggesting a contribution of neuroinflammation in the pathogenesis of AD. Besides cognitive deterioration, non-cognitive symptoms, such as agitation, aggression, depression and psychosis, are often observed in demented patients, including those with AD, and these neuropsychological symptoms place a heavy burden on caregivers. These symptoms often exhibit sudden onset and tend to fluctuate over time, and in many cases, they are triggered by an infection in peripheral organs, suggesting that inflammation plays an important role in the pathogenesis of these non-cognitive symptoms. However, there is no mechanistic explanation for the relationship between inflammation and neuropsychiatric symptoms. Observations from experimental mouse models indicate that alteration of brain blood vessels, especially blood-brain barrier (BBB) dysfunction, may contribute to the relationship. The current review summarizes the results from recent studies on the relationship between inflammation and AD, while focusing on cerebrovascular alterations, which might provide an insight into the pathogenesis of cognitive/non-cognitive symptoms in AD patients and suggest a basis for the development of new therapeutic treatments for these conditions.

## Introduction

Alzheimer disease (AD) is the most common form of dementia, accounting for around 60–90% of all cases (Thies and Bleiler, 2013), and its incidence is increasing at an alarming rate all over the world. AD is characterized by progressive cognitive and behavioral impairment, and cerebral deposition of senile plaques (extracellular accumulation of beta-amyloid (Aβ) peptide) and neurofibrillary tangles (NFTs, intracellular accumulation of hyperphosphorylated tau protein) are unique neuropathological hallmarks of the disease (Hardy and Selkoe, 2002; Iqbal and Grundke-Iqbal, 2006). Multiple so-called “disease-modifying” therapies for AD, such as immunotherapy against Aβ, are now under investigation and have been tested in clinical trials; however, most of them have had little success so far (Salloway et al., 2008; Delrieu et al., 2012; Thies and Bleiler, 2013). There is an urgent need to identify novel targets to develop alternative therapeutic strategies.

There is a growing body of evidence linking inflammation and the pathogenesis of AD (Akiyama et al., 2000; Lee et al., 2010; Holmes and Butchart, 2011). Microglia, which play a critical role in the brain immune system, are known to be activated in the AD brain, and clusters of reactive microglia are found associated with amyloid plaque (Serrano-Pozo et al., 2013). Recently, it has been reported that CD33, which regulates the innate immune system, as well as its gene, has been shown to be a risk factor for AD, CD33 inhibits clearance of Aβ by microglia, and deletion of CD33 in an Alzheimer transgenic mouse significantly reduced pathological amyloid in the brain (Bradshaw et al., 2013; Griciuc et al., 2013). Although the role of neuroinflammation in the AD brain remains unclear, findings from experimental disease models and clinical studies have demonstrated a significant contribution of inflammation to pathological features and symptoms of AD.

On the other hand, recent epidemiological studies have provided direct evidence linking AD and vascular risk factors such as hypertension and diabetes mellitus, which is now attracting attention (Takeda et al., 2008; Sato et al., 2011; Sato and Morishita, 2013). Also, it is well recognized that the pathogenesis of AD and vascular dementia overlap (Iadecola, 2010, 2013; Muresanu et al., 2014). Cerebrovascular alteration, which is a common pathological change in these diseases, is a possible mechanism of this relationship. Our recent studies using unique AD mouse models showed that cerebrovascular inflammation induced by a diabetic condition (Takeda et al., 2010) or peripheral inflammation (Takeda et al., 2013) had a significant impact on pathological features of the AD brain and cognitive/non-cognitive symptoms of the animals.

In this review, we discuss recent findings on the relationship between inflammation and AD, focusing on cerebrovascular alterations, which might provide a new insight into the pathogenesis of AD and a basis for the development of an alternative therapeutic strategy for the disease.

## AD neuropathology and inflammation

In addition to classical neuropathological signs like amyloid plaques and NFT, significant accumulation and infiltration of activated immune cells have been well documented in the AD brain (Dickson et al., 1993; Akiyama et al., 2000; Letiembre et al., 2009), suggesting a contribution of neuroinflammation to AD pathogenesis. Clustering of activated microglia (Haga et al., 1989; Simard et al., 2006) and astrocytes (Mrak et al., 1996) around amyloid plaques is observed in the AD brain, associated with upregulated expression of a variety of inflammatory cytokines, including interleukin (IL)-1 (Griffin et al., 1995), IL-6 (Bauer et al., 1991), *tumor necrosis factor-α* (TNF-α; Tarkowski et al., 1999), and transforming growth factor-β (TGF-β; van der Wal et al., 1993). It has been shown that innate immune receptors, such as toll-like receptor and lipopolysaccharide (LPS) receptor (CD14), are upregulated in the human AD brain (Letiembre et al., 2009). Furthermore, it is well known that most of the AD transgenic mouse models exhibit significant enhancement of neuroinflammation around amyloid plaques, like those in the human AD brain (Frautschy et al., 1998; Matsuoka et al., 2001; Janelsins et al., 2005), and its severity increases with progression of pathological amyloid with age (Sheng et al., 2000). Importantly, some markers of glial activation are elevated even before the development of amyloid deposition, implying that neuroinflammation precedes typical AD neuropathological changes and has some causative effect in AD pathogenesis (Sheng et al., 2000). Recent high-resolution positron emission tomographic (PET) imaging has enabled longitudinal observation of amyloid accumulation and associated microglial activation in the Alzheimer amyloid precursor protein (APP) transgenic mouse, and demonstrated a dynamic change in microglial activity in response to anti-amyloid therapy (Maeda et al., 2007; Ji et al., 2008).

However, whether activation of the local immune system in the diseased brain is the cause or consequence of AD, and whether it is causes a positive or negative effect on disease progression remain controversial. It has been reported that direct injection of IL-1 into the rodent brain upregulates beta-APP, supporting the idea that overexpression of IL-1 in the brain could be a driving force in AD pathogenesis (Sheng et al., 1996). Craft et al. (2005) reported that IL-1 receptor antagonist knockout mice, which have enhanced IL-1 signaling, show increased vulnerability to Aβ-related toxicity, suggesting that enhanced neuroinflammation mediated by IL-1 could have an unfavorable effect on the AD brain. On the other hand, Shaftel et al. (2007) have shown that local overexpression of IL-1β in the hippocampus of an APP transgenic mouse model significantly decreased the severity of pathological amyloid, demonstrating a possible adaptive role of IL-1β in AD pathogenesis. Similarly, an APP transgenic mouse model overexpressing IL-6 in the brain showed attenuated Aβ deposition associated with enhanced gliosis, indicating that IL-6-mediated gliosis could have a favorable effect in terms of clearing Aβ from the brain (Chakrabarty et al., 2010). However, Ghosh et al. (2013) also reported that sustained IL-1β overexpression exacerbated pathological tau despite reduced Aβ burden in an AD mouse model. These conflicting findings suggest a complex role of inflammation in the AD brain.

Along the same line, the therapeutic effect of anti-inflammatory drugs on human AD patients is still controversial (McGeer et al., 1996). Findings from experimental studies using mouse model have shown favorable effects of anti-inflammatory agents such as nonsteroidal anti-inflammatory drugs (*NSAIDs*) on AD neuropathological features and cognitive disturbance (Lim et al., 2000, 2001; Yan et al., 2003). However, clinical trials of traditional anti-inflammatory drugs, including *NSAIDs* (Breitner et al., 1994; Rich et al., 1995) and cyclooxygenase (COX) inhibitors (McGeer, 2000; Trepanier and Milgram, 2010), have failed to demonstrate their positive effect on AD patients (Rogers et al., 1993; Scharf et al., 1999; Aisen et al., 2003; Reines et al., 2004), though it has been claimed that they have lower accessibility into the brain.

## Systemic inflammation and AD

The brain has been considered to be an “immune-privileged” organ, isolated from the peripheral immune system (Shrestha et al., 2013). However, recent evidence shows that there is bi-directional communication between the brain and the peripheral immune system (Holmes and Butchart, 2011). A neural route via the vagal nerve, circumventricular organs that lack a blood-brain barrier (BBB), direct entry of monocytes through the BBB, and inflammatory lipid mediators such as prostaglandins that can cross the BBB are recognized to be important communication pathways connecting the brain and the systemic immune system (Quan and Banks, 2007). Alteration of peripheral immune activity can affect the brain immune system through these pathways. In the AD brain, the BBB becomes leaky due to damage from accumulation of Aβ along brain blood vessels (*cerebral amyloid angiopathy*, CAA) and associated vascular inflammation (Greenberg et al., 2004; Kinnecom et al., 2007), which could alter communication between the brain and the peripheral immune system. Activated peripheral immune cells that enter the brain though the leaky BBB may directly or indirectly affect the central nervous immune system and modulate the pathogenesis of AD. Indeed, Dunn et al. (2005) reported an association between systemic inflammation and increased risk of dementia in a case-control study. Although clinical evidence linking the risk of developing AD and systemic inflammation is still limited and controversial (Sundelof et al., 2009), some observational studies and a meta-analysis showed that elevated peripheral inflammatory markers are associated with increased risk of overall dementia (Koyama et al., 2013), suggesting a positive correlation between systemic inflammation and neurodegeneration.

This interaction between the peripheral immune system and the brain might provide a possible explanation for the linkage of some chronic diseases like hypertension in midlife and diabetes, in which persistent inflammation plays a significant role in their pathogenesis, with increased risk of dementia including AD.

## Inflammation and dementia-associated behavioral disturbance

Non-cognitive symptoms, such as agitation, aggression, depression and psychosis, are often observed in demented patients including those with AD, in addition to progressive cognitive deterioration. These symptoms, known as “behavioral and psychological symptoms of dementia” (BPSD), have been reported to occur in about 20% of AD patients (Lyketsos et al., 2000) and affect up to 80% of patients living in social care facilities and nursing homes (Margallo-Lana et al., 2001; Ryu et al., 2005), placing an extremely heavy burden on families and caregivers (Corbett et al., 2012; Ballard and Corbett, 2013). These neuropsychological symptoms often exhibit sudden onset and tend to fluctuate over time. In many cases, they are triggered by an acute change in the patient’s physical condition such as dehydration, pain, or exacerbation of an existing chronic health condition. Infection in peripheral organs, such as pneumonia or urinary tract infection, is often associated with manifestation of BPSD (Holmes and Butchart, 2011), suggesting that inflammation could play an important role in the pathogenesis underlying these dementia-associated behavioral disturbances. Importantly, severe neuropsychological symptoms triggered by peripheral infection can develop without direct bacterial invasion into the brain spreading from peripheral organs (van Gool et al., 2010) and without signs of sepsis (Ebersoldt et al., 2007). A prospective matched control study has shown a strong association between dementia and postoperative delirium in elderly people who underwent hip surgery, suggesting a link between systemic inflammation induced by peripheral intervention and dementia-associated psychiatric symptoms (Kat et al., 2008). However, at present, specific mechanisms for the susceptibility of demented patients to infection-triggered neuropsychological symptoms have not been proposed.

Some experimental findings using animal models have suggested possible mechanisms for the effect of systemic infection on behavioral disturbance. Acute and chronic infusion of LPS into the rat brain impaired the function of cholinergic neurons, which is essential for maintaining healthy brain function, associated with accumulation of reactive astrocytes and microglia within the basal forebrain (Willard et al., 1999). Godbout et al. reported that LPS-induced peripheral inflammation caused more severe neuroinflammation and associated behavioral disturbances in elderly mice compared to young mice, which indicates that an age-related potential augmentation of neuroinflammatory response might affect the susceptibility to behavioral problems following activation of the peripheral immune system (Godbout et al., 2005; Godbout and Johnson, 2009). The Alzheimer APP transgenic mouse is known to develop a variety of behavioral problems such as diurnal rhythm disturbance and increased aggression resembling BPSD in demented patients (Vloeberghs et al., 2004, 2007), which could be a valuable tool for investigating more specific mechanisms of the neuropsychological symptoms in AD.

## Blood-brain barrier, cerebrovascular function and neurodegenerative diseases

The entry of most molecules into the central nervous system from the peripheral circulation is strictly regulated by the BBB, and its function and integrity are maintained by the “neurovascular unit”, which is composed of vascular endothelial cells, astrocytes, pericytes (Hall et al., 2014), and neurons (Bell and Zlokovic, 2009; Neuwelt et al., 2011; Zlokovic, 2011). Brain endothelial cells lining cerebral microvessels act as a physical barrier because they are sealed together by tight junctions, resulting in limited paracellular diffusion, and they have no fenestrations, in contrast to leaky vessels in peripheral organs (Abbott et al., 2006; Ricci et al., 2006). These endothelial cells are surrounded by a basal lamina and the endfeet of astrocytes, which contribute to BBB function and integrity by regulating expression levels of tight junction molecules (Dehouck et al., 1990) and specific transporters (McAllister et al., 2001). Interactions between endothelial and astroglial cells at the BBB play an important role in maintaining proper brain function by regulating regional cerebral blood flow and metabolism, which could affect local neuronal function (Abbott et al., 2006).

There is emerging evidence that BBB dysfunction is associated with the pathogenesis of a variety of neurodegenerative disorders such as AD (Bell and Zlokovic, 2009; Zlokovic, 2011), Parkinson disease (Chung et al., 2010; Bartels, 2011), multiple sclerosis (Lassmann et al., 2012; Prineas and Parratt, 2012; Tourdias and Dousset, 2013), and amyotrophic lateral sclerosis (Rodrigues et al., 2012; Garbuzova-Davis and Sanberg, 2014; Winkler et al., 2014), in addition to typical cerebrovascular disorders such as stroke and vascular dementia (Wardlaw et al., 2003; Iadecola, 2013).

It has become recognized that there is significant overlap between the pathogenesis of AD and that of vascular dementia. The results of recent epidemiological studies have shown that vascular risk factors such as hypertension (Skoog and Gustafson, 2003), diabetes mellitus (Ott et al., 1999; Sato et al., 2011) and atherosclerosis (Takeda et al., 2008) have a significant impact on the progression of AD, although the mechanisms underlying these interactions remain unclear. One of the possible explanations for this association is that cerebrovascular damage caused by these diseases could affect cognitive function. Gentile et al. (2009) examined the effect of hypertension on pathological amyloid in the brain using two mouse models of hypertension, demonstrating Aβ deposition in the hypertensive mouse brain which was associated with impairment of BBB integrity. It has been reported that some antihypertensive drugs restored cerebrovascular dysfunction via reduction of oxidative stress and improved cognitive function in an AD mouse model (Takeda et al., 2009). Our previous study demonstrated that, using unique diabetic AD mouse models, co-existence of a diabetic condition could accelerate Aβ-related vascular alterations via increased expression of inflammatory cytokines, which was associated with induction of the receptor for advanced glycation end products (RAGE) in the cerebral vasculature (Takeda et al., 2010). These findings imply that cerebrovascular dysfunction plays a role not only in vascular dementia but also in the AD brain with non-genetic risk factors.

*ApoE*-ε4 is a strong genetic risk factor for sporadic AD; however, how it impacts on AD pathogenesis is still unknown (Bu, 2009). The *APOE*-ε4 allele increases the accumulation of senile plaques in AD patients as well as in cognitively normal people (Reiman et al., 2009; Morris et al., 2010). One recent report has shown that ApoE also regulates cerebrovascular function via the cyclophilin A (CypA)-nuclear factor-κB (NF-κB)—matrix-metalloproteinase-9 (MMP9) pathway in pericytes in an isoform-specific manner, and that CypA could be a target for treating *ApoE*-ε4-mediated neurovascular impairment and the related neuronal malfunction (Bell et al., 2012). This observation could provide a new insight for understanding the cerebrovascular-related pathogenesis of AD, linking the contribution of *ApoE*-ε4 as a risk factor.

## BBB function in Alzheimer brain

Some studies have shown that BBB integrity is compromised in Alzheimer APP transgenic mice even before they develop amyloid plaques and cognitive impairment. Ujiie et al. (2003) examined BBB integrity in the Tg2576 AD mouse model using a well-established method based on dye leakage, and found that AD mice exhibit breakdown of the BBB as early as 4 months of age, which is much earlier than the manifestation of amyloid plaque deposition and cognitive dysfunction. This observation implies that functional alteration of the BBB could be one of the earliest signs of AD. Along the same line, Dickstein et al. (2006) demonstrated that Aβ immunization therapy could restore BBB integrity in the same AD mouse model (Tg2576), in addition to improvement of typical AD brain pathological features such as plaque burden and microgliosis, suggesting a new strategy for AD treatment focusing on BBB function (Dickstein et al., 2006). However, we need to give careful consideration to the effect of Aβ immunization on BBB function because it has also been demonstrated that passive immunization with Aβ antibodies resulted in a significant increase in both the frequency and severity of CAA-related microhemorrhages in different APP transgenic mouse models (Pfeifer et al., 2002; Racke et al., 2005; Burbach et al., 2007; Luo et al., 2010).

Our recent study has shown that, using an Alzheimer APP transgenic mouse, peripheral inflammation could be more likely to spread into the brain due to increased vulnerability of the blood-brain-barrier to peripherally evoked inflammation, leading to more severe non-cognitive symptoms (Takeda et al., 2013). After peripheral injection of LPS to induce systemic inflammation, APP transgenic mice showed a greater increase in inflammatory cytokine levels in brain interstitial fluid and more severe non-cognitive symptoms (such as attenuation of basal activity, social interaction behavior, and food intake) compared to wild-type control animals, while there was no difference in inflammatory cytokine levels in plasma and peripheral organs between genotypes. The BBB became leaky in the AD transgenic mouse brain during the course of inflammation, suggesting increased vulnerability of the BBB to systemic inflammation in the AD mouse brain, which might be a mechanism for peripheral inflammation spreading more easily to the brain. Importantly, no clear change in brain Aβ level was observed after peripheral administration of LPS, meaning that the behavioral symptoms induced by peripherally evoked inflammation may not be due to change in brain Aβ level. These findings indicate that the potential vulnerability of the BBB to inflammation underlies the increased severity of behavioral impairment in AD.

The detailed mechanisms of BBB breakdown during systemic inflammation in the AD mouse brain remain unclear and need to be elucidated in order to tackle this pathological event. The relationship between Aβ and endothelial cells has been under extensive investigation and there is substantial evidence that Aβ could alter endothelial function, which plays a critical role in BBB integrity (Suhara et al., 2003; Vagnucci and Li, 2003; Hayashi et al., 2009; Takeda et al., 2009). It has been demonstrated *in vitro* that intracellular Aβ42 could be toxic to endothelial cells via disruption of the Akt/glycogen synthase kinase (GSK)-3β pathway (Suhara et al., 2003). Hayashi et al. (2009) also reported that Aβ40 altered endothelial function and impaired vascular regeneration through the induction of autophagy. Another report showed that oxidative stress was increased in brain blood vessels of an APP transgenic mouse (APP23 mouse) even before the manifestation of amyloid plaques and CAA (Takeda et al., 2009). These changes in the cerebral vasculature might potentially increase the vulnerability of the BBB in the AD brain.

## Summary

**Figure 1:**
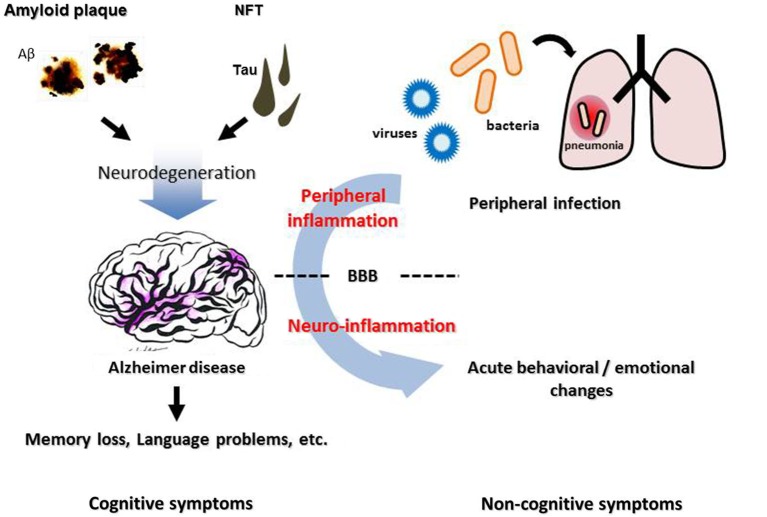
**Neuropathological features, inflammation, and cognitive/non-cognitive symptoms of Alzheimer disease**. Cerebral accumulation of amyloid plaques and neurofibrillary tangles (NFT) leads to neurodegeneration in the AD brain, which causes progressive cognitive dysfunction such as memory loss and language problems. Non-cognitive symptoms, such as agitation, aggression and psychosis, are often observed in AD patients, besides cognitive deterioration. These symptoms can be triggered by an infection in peripheral organs such as pneumonia, suggesting a contribution of peripheral inflammation. BBB, blood-brain barrier.

A growing body of evidence links systemic inflammation and AD pathogenesis in the brain. Although the overall impact of the activated brain immune system on AD pathological features is complicated and still controversial, it is clear that it could be affected by the peripheral immune system. Direct or indirect modulation of the brain immune system from the periphery could be a new strategy to alter AD progression. The cerebral vasculature, especially the BBB, is an important interface linking peripheral inflammation and the AD brain (Figure 1). It remains unclear how exactly systemic inflammation could damage the integrity and function of the BBB and alter cognitive function, and the mechanisms need to be elucidated in order to develop new treatments for AD focusing on vascular and other interface pathology.

Moreover, there is a clear cause-and-effect relationship between activated systemic inflammation and the development of neuropsychiatric symptoms in AD, although no mechanistic explanation for the relationship exists thus far. Furthermore, to date, there is no alternative treatment for non-cognitive symptoms of dementia directed against specific pathological mechanisms. Inflammation-related cerebrovascular alterations such as BBB breakdown could be a potential target for new therapies tackling those symptoms.

## Conflict of interest statement

The authors declare that the research was conducted in the absence of any commercial or financial relationships that could be construed as a potential conflict of interest.
